# Blood pressure regulation V: in vivo mechanical properties of precapillary vessels as affected by long-term pressure loading and unloading

**DOI:** 10.1007/s00421-013-2758-9

**Published:** 2013-12-07

**Authors:** Ola Eiken, Igor B. Mekjavic, Roger Kölegård

**Affiliations:** 1 Department of Environmental Physiology and Swedish Aerospace Physiology Centre, KTH, Royal Institute of Technology, Berzelius v 13, Solna, 17165 Stockholm, Sweden; 2 Department of Automation, Biocybernetics and Robotics, Jozef Stefan Institute, Ljubljana, Slovenia

**Keywords:** Arterial compliance, Hypotension, Peripheral blood-flow resistance, Vascular remodelling, Vascular deconditioning, Vascular wall tension

## Abstract

Recent studies are reviewed, concerning the in vivo wall stiffness of arteries and arterioles in healthy humans, and how these properties adapt to iterative increments or sustained reductions in local intravascular pressure. A novel technique was used, by which arterial and arteriolar stiffness was determined as changes in arterial diameter and flow, respectively, during graded increments in distending pressure in the blood vessels of an arm or a leg. Pressure-induced increases in diameter and flow were smaller in the lower leg than in the arm, indicating greater stiffness in the arteries/arterioles of the leg. A 5-week period of intermittent intravascular pressure elevations in one arm reduced pressure distension and pressure-induced flow in the brachial artery by about 50 %. Conversely, prolonged reduction of arterial/arteriolar pressure in the lower body by 5 weeks of sustained horizontal bedrest, induced threefold increases of the pressure-distension and pressure-flow responses in a tibial artery. Thus, the wall stiffness of arteries and arterioles are plastic properties that readily adapt to changes in the prevailing local intravascular pressure. The discussion concerns mechanisms underlying changes in local arterial/arteriolar stiffness as well as whether stiffness is altered by changes in myogenic tone and/or wall structure. As regards implications, regulation of local arterial/arteriolar stiffness may facilitate control of arterial pressure in erect posture and conditions of exaggerated intravascular pressure gradients. That increased intravascular pressure leads to increased arteriolar wall stiffness also supports the notion that local pressure loading may constitute a prime mover in the development of vascular changes in hypertension.

## Introduction

For decades, it has been recognized that the mechanical properties of peripheral blood vessels play key roles in circulatory control, both in physiological and pathological conditions (for reviews see Folkow and Neil 1971; Folkow 1982, 1990; Dobrin 1983; Intengan and Schiffrin 2000), a fact that, one way or the other, is also acknowledged in several of the articles included in the present thematic series of reviews on arterial pressure regulation (see for instance: Ichinose et al. 2013; Joyner and Limberg 2013; Padilla et al. 2013). Notwithstanding, until recently, firm data have been scarce regarding the issues discussed in the present review, namely, the in vivo stiffness—and its inverse, distensibility—of precapillary vessels in humans, and in particular how this property adapts to iterative increments or sustained reductions in local intravascular pressure.

Because the luminal cross-sectional area of the arterioles determines flow resistance, it is evident that the passive elastic recoil and myogenic tone of precapillary vessels constitute important elements in the control of arterial pressure. It is perhaps less obvious whether, or in what manner, changes in the prevailing transmural pressure will, in turn, affect the tone and/or structure, and hence the elastic recoil, of these vessels. In healthy humans, sustained changes in arterial and arteriolar wall tension occur regularly, not only as a consequence of systemic alterations of arterial pressure, such as in connection with altered level of physical or mental stress, but also due to local pressure changes induced by the gravitoinertial force field. Thus, in erect posture, gravitational pull causes marked hydrostatic pressure components in dependent blood vessels. In a pilot flying a high-performance aircraft, the force vector acting in the head-to-foot direction may be increased several-fold, resulting in greatly exaggerated intravascular pressure gradients (Burton and Whinnery 2008). The force vector acting along longitudinally-oriented vessels may, by contrast, be abolished or minimized by assuming a horizontal body position or, more drastically, by exposure to microgravity. Conceivably, such long-lasting or reoccurring alterations of local transmural pressure will influence the pressure-distension relationships of arteries and arterioles.

**Fig. 1:**
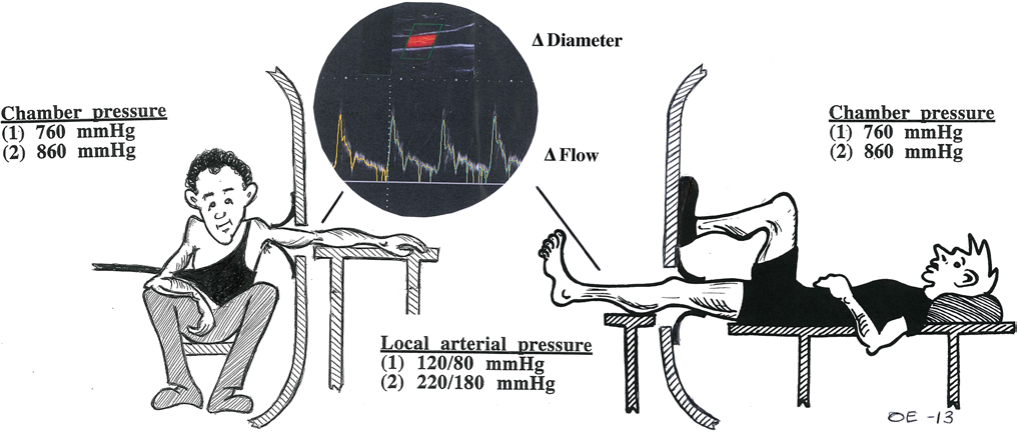
The cartoons depict the experimental set-ups employed to induce graded increments of the distending (transmural) pressure in the vascular beds of an arm or a leg. The subject is positioned inside a hyperbaric chamber with the test limb extended to the outside via a hole in the chamber door. As chamber pressure is raised, distending pressure in the arteries and arterioles of the test limb increases in direct proportion to the applied pressure, in the these examples by 100 mmHg from normal atmospheric pressure (760 mmHg); see also in the text under “Introduction”

To establish the pressure-distension relationships in human peripheral blood vessels, changes in diameter and/or flow should be investigated across a wide range of distending pressures. The concept that the mechanical properties of vascular walls adapt to cope with changes in the pressure load is not novel (for reviews see Folkow 1982, 1990; Dobrin 1983; Intengan and Schiffrin 2000), but due to the practical problems associated with accurately measuring vascular flow and diameter responses to graded changes in the distending pressure in vivo, information is scarce regarding arterial/arteriolar pressure-distension relationships in humans. The present review is largely based on a series of experimental studies (Eiken et al. 2008, 2011, 2012; Eiken and Kölegård 2001, 2004, 2011; Gustafsson et al. 2013), three of which have been summarized previously (Kölegård 2010). In these studies, the pressure distension of arteries and arterioles in the extremities of healthy humans were examined using a technique described in detail elsewhere (Eiken and Kölegård 2001, 2004). Briefly, the subject is positioned inside a hyperbaric chamber with either an arm or a lower leg (test limb) protruding to the outside via a hole in the chamber door (Fig. 1). The test limb is sealed to the door hole slightly distally of the axilla, or proximally of the knee, by use of a short, loose-fitting, self-sealing sleeve. As chamber pressure is elevated, pressure increases in tissues enclosed in the chamber and is also transmitted to the blood vessels of the test limb outside the chamber. However, pressure in the extravascular tissues of the protruding test limb is similar to ambient pressure outside the chamber. In this manner, the distending (transmural) pressure in the test-limb vasculature can be elevated to any desired level, and distension of arteries can be determined directly using ultrasonographic techniques, whereas arteriolar distension can be determined indirectly via measurement of pressure-induced changes in flow by means of ultrasound/Doppler techniques. Distending pressure of the test-limb arteries can be established by measuring arterial pressure, relative to ambient pressure outside the chamber, either in the test limb or in an arm or a finger inside the chamber, whereas, the average arteriolar distending pressure of the test limb is not readily determined. Therefore, in the following also arterial flows are treated as functions of arterial pressure, assuming that peak arteriolar distending pressure at the upstream end of the arterioles, corresponds to arterial distending pressure.

In this connection, it should be noted that in vivo distensibility of human conduit arteries are commonly approximated by tracking dynamic changes in the vessel diameter during the course of a pulse-pressure wave (Nichols 2005; Cinthio et al. 2010). Such measurements can readily be performed in vivo, in healthy individuals as well as in patients, and their estimates may, under certain conditions, serve as markers of arterial distensibility, and hence these techniques constitute useful tools to evaluate various cardiovascular pathologies, including risk prediction of coronary disease and of mortality in hypertensive patients (cf. Laurent et al. 2001; Nichols 2005; Kips et al. 2012). Nevertheless, they do not reveal the true distension of peripheral arteries and arterioles during marked static increases of local transmural pressure; as evident from the following sections, these relationships describe non-linear functions with significant arterial/arteriolar distension occurring only at markedly elevated transmural pressures. In our experience, it is not fruitful to apply techniques based on analyses of conduit artery diameter changes during pulse-pressure waves in a supine resting human, to study how interventions, comprising iterative increments or sustained reductions of static transmural pressures, affect local distensibility of arteries and arterioles.

## Arterial pressure-distension and pressure-flow relationships in the arm compared to in the leg

**Fig. 2:**
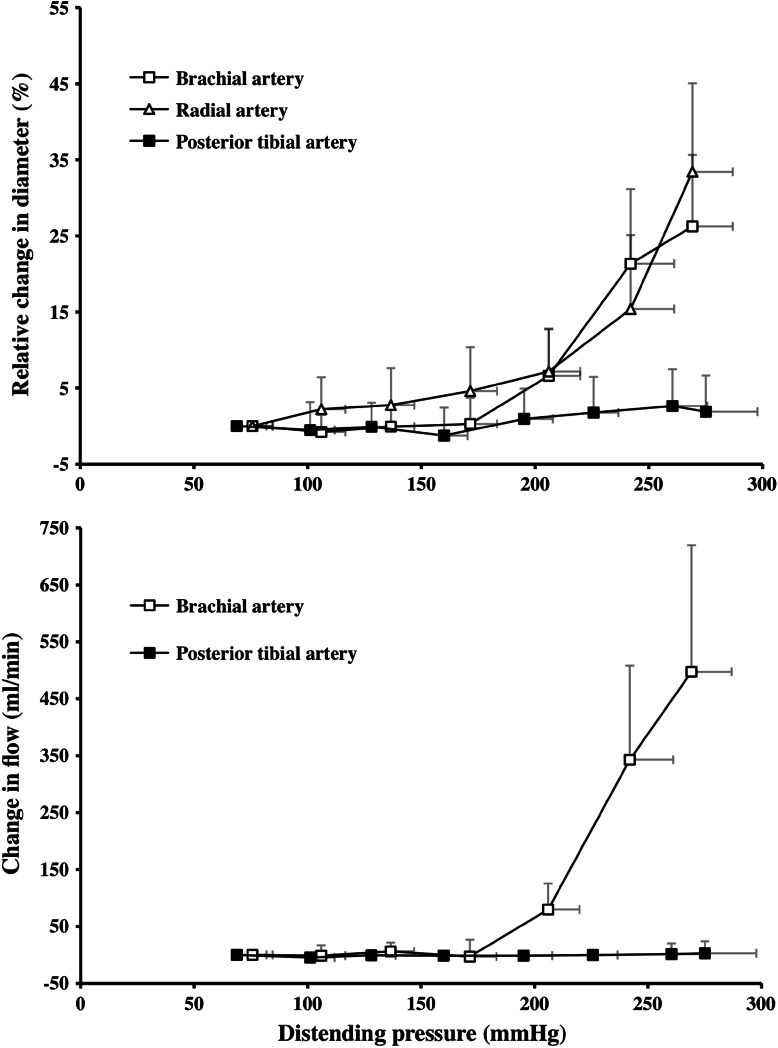
Relative change in the lumen diameter (*upper panel*) of the brachial artery, radial artery and posterior tibial artery, and of the change in flow (*lower panel*) in the brachial and posterior tibial arteries as functions of distending pressure. Values are means (SD). *n* = 10 for *upper panel* and *n* = 7 for *lower panel*. Adapted from Eiken and Kölegård (2004)

In erect posture, gravity-dependent hydrostatic pressure components exert substantial influence on local arterial pressure, so that in a normotensive adult, diastolic pressure is 70–100 mmHg higher in the tibial arteries, at ankle level, than in the distal brachial arteries. To test the hypothesis that arterial and arteriolar stiffness adapts to meet the long-term demands imposed by the local hydrostatic pressure (cf Folkow 1982; Dobrin 1983), pressure-distension and pressure-flow relationships were compared in arm and leg arteries of 10 healthy individuals (Eiken and Kölegård 2004; Fig. 2). Brachial and radial artery diameters, as well as the brachial artery flow, remained virtually unaltered at slight to moderate elevations of the distending pressure, whereas at markedly elevated pressures (>160–170 mmHg) the diameters and flows increased substantially in the arm arteries. Tibial artery diameter and flow, on the other hand, did not show any significant changes at elevations of the distending pressure up to about 275 mmHg (Fig. 2). Greater pressure-distension and pressure-flow responses in the arm than in the lower leg are compatible with the notion that the in vivo stiffness of arteries and arterioles will adapt to cope with the local pressure perturbations associated with everyday life in erect posture. That precapillary vessels in the legs are stiffer than those in the arms is also suggested by the combined results from studies in which changes in skin blood flow were approximated in the hand (Coles and Greenfield 1956) and toes (Coles 1957) using a calorimetric method, and from a study on regional differences in arterial pulse-wave velocity (McDonald 1968).

A central question is whether the differences between the arm and leg vessels as regards pressure-distension relationships are attributable to functional or structural features. It is well established from experiments on animals that sustained arterial pressure loading initially leads to increased myogenic tone in small arteries and arterioles; depending on the magnitude and duration of the pressure load, this functional adjustment may be followed by structural wall changes, manifesting themselves as increased passive recoil (for reviews see Folkow 1982, 1990). It remains to be established whether the iterative gravity-dependent pressure elevations in leg arteries/arterioles associated with a normal ambulatory life style are sufficient to affect not only the myogenic tone but also the elastic recoil properties of the vessels.

**Fig. 3:**
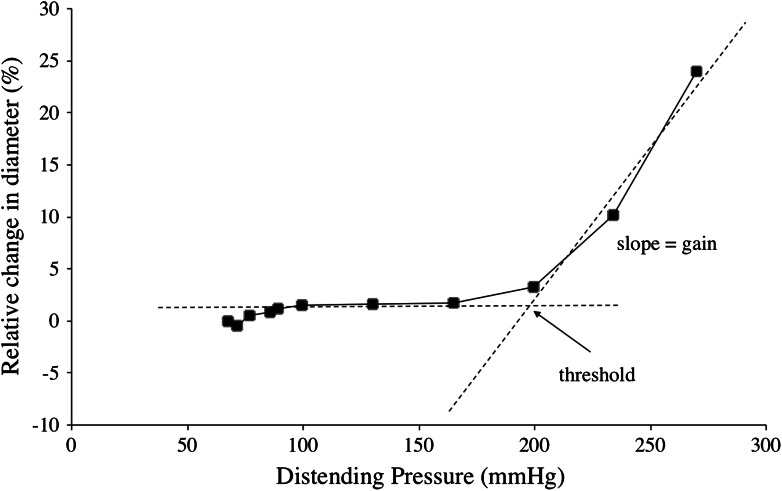
Principles for the analyses of threshold and gain of the pressure-diameter and pressure-flow relationships explained in the text. The pressure-distension curves (and pressure-flow curves; not depicted) can be described by identifying the pressure threshold and gain (slope) of the curve. *n* = 8. Values are from Eiken and Kölegård (2001)

Similar in vivo non-linear pressure-diameter and pressure-flow relationships, as those found for the brachial and radial arteries (Fig. 2), have also been observed in animals, the shapes of the curves being typical for arteries with preserved myogenic tone (Folkow et al. 1970a, b; Folkow 1982). The flat portion of the curve, at low distending pressures, represents the phase during which arterial/arteriolar lumen diameters are maintained via autoregulation of the myogenic tone of the vessel walls (cf. Folkow and Sivertsson 1968; Folkow et al. 1970a, b, Folkow 1982); at high distending pressures, where the myogenic tone is not sufficient to prevent an increase in diameter, the slopes of the pressure-distension curves are predominantly determined by the elastic recoil properties of the wall (cf. Folkow and Sivertsson 1968; Folkow et al. 1970a, b; Folkow 1982), as depicted schematically in Fig. 3. Considering the aforementioned pressure-diameter and pressure-flow relationships (Fig. 2) in this perspective, it appears clear that the arteries and arterioles of the lower leg are characterized by a considerably higher myogenic tone than those of the arm. In the experiments by Eiken and Kölegård (2004), the distending pressure was not elevated sufficiently to reveal the gains for the pressure increments in tibial artery diameter and flow, but subsequent experiments (Eiken et al. 2008), showed that also the gain for the pressure-induced increase in diameter was greater in the brachial artery than in the tibial artery (Table 1), which might suggest that not only the myogenic tone but also the passive elastic recoil is higher in the leg arteries/arterioles.

**Table 1:** Pressure-diameter relationships in the brachial and posterior tibial arteries in terms of the pressure thresholds and gains for the pressure-induced diameter increments

	Brachial artery	Tibial artery
Diameter		
Threshold (mmHg)	168 ± 19**	250 ± 31
Gain (∆ %/mmHg)	0.35 ± 0.19**	0.21 ± 0.16

** Denotes difference (*p* < 0.01) between brachial and tibial artery. *n* = 10. Values are based on results obtained from Eiken et al. (2008). Values are mean ± SD

There seem to be no other experimental studies determining in vivo regional differences in arterial and arteriolar elastic recoil properties in humans, whereas there are several studies supporting the notion that the myogenic tone in precapillary vessels is higher in the leg than in the arm. For instance, the relative increase in arterial flow following local infusion of vasodilators is greater in the arm than in the leg (Newcomer et al. 2004), and sympathetically-mediated vasoconstrictor responses are more pronounced in the legs than in the arms (Pawelczyk and Levine 2002). Likewise, endothelium-dependent vascular reactivity is greater in the leg than in the arm (Nishiyama et al. 2008).

It appears that both arterial and arteriolar pressure distension in the arm occur at about 160–180 mmHg (Fig. 2), i.e. at magnitudes prevailing in the lower leg in erect posture. Thus, it seems reasonable to assume that the high wall stiffness in the leg arterioles serves to prevent a drop in local peripheral resistance upon a change in body position from recumbent to upright, and hence constitutes an important adaptation to life in erect posture.

That the capacity of leg arterioles to withstand transmural pressure elevations plays an important role in the control of arterial pressure under conditions of increased hydrostatic pressure gradients, is also supported by findings from experiments in a human-use centrifuge; during exposure to gradually increasing gravitoinertial (G) load in the head-to-foot direction, individuals with high stiffness in lower-leg arterioles maintain arterial pressure at heart level more efficiently, and hence tolerate substantially higher G loads, than do individuals with low stiffness in the arterioles of the lower legs (Eiken et al. 2012). Analyses of thresholds and gains of the pressure-diameter and pressure-flow relationships (see text above and Fig. 3), suggest that the inter-group differences in precapillary stiffness are attributable mainly to differences in local myogenic tone, and only to a lesser degree to differences in passive elastic recoil (Eiken et al. 2012).

Notably, the observation that the stiffness in arteries and arterioles is higher in the legs than in the arms in healthy individuals, does not give any indication as to whether these properties are plastic or if lifelong exposure to gravity-induced local pressure perturbations is required to induce such differences. It also remains to be established whether inter-individual differences in the pressure-distension relationships of arteries and arterioles are permanent or are the result of differences in the prevailing pressure or other conditioning factors.

## Arterial pressure-distension and pressure-flow relationships as affected by repeated increments of transmural pressure

The notion that the wall stiffness of precapillary resistance vessels increases in response to sustained or iterated elevations of arterial pressure was introduced more than a century ago by Ewald (1877), who based his theory on the arteriolar media hypertrophy observed in patients suffering from Brights disease. Ever since, it has repeatedly been postulated that such a mechanism may play a pivotal role in the development of primary hypertension (for reviews see Pickering 1977; Folkow 1982, 1990; Intengan and Schiffrin 2000). Indeed, cross-sectional studies have shown evidence to suggest, indirectly (Folkow et al. 1958) and directly (Turnbull 1915; Aalkjaer et al. 1987; Prewitt et al. 2002), that individuals suffering from chronic hypertension exhibit increased wall thickness-to-lumen ratio in small arteries and resistance vessels, structural changes that will increase the flow resistance for any given smooth muscle activity (Folkow et al. 1958; Folkow and Sivertsson 1968; Folkow 1990). In a physiological context, a mechanism by which the stiffness of resistance vessels increase in response to markedly increased pressure loading may serve to protect the capillary beds and venules from downstream propagation of excessive arterial pressure elevations. That changes in the prevailing transmural pressure may also affect the in vivo mechanical properties of precapillary resistance vessels, is supported by experimental findings on animals, that long-term intravascular pressure elevations increase local blood-flow resistance (for reviews see Folkow 1982, 1990; Intengan and Schiffrin 2000; Zhang 2001).

**Fig. 4:**
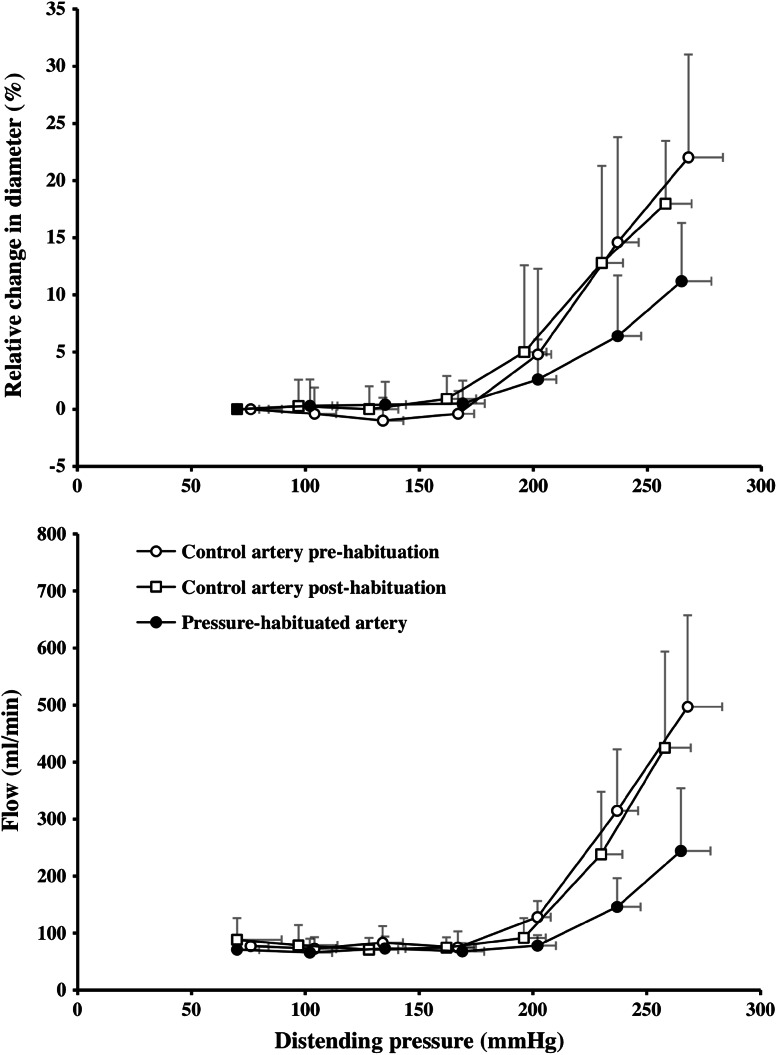
Relative change in the lumen diameter (*upper panel*) and flow (*lower panel*) in the brachial artery as functions of distending pressure, in the control artery (unhabituated arm) before and after the 5-week pressure-habituation regimen and in the pressure-habituated artery following the pressure habituation. Values are means (SD). *n* = 11 for *upper panel* and *n* = 7 for *lower panel*. Adapted from Eiken and Kölegård (2011)

As regards humans, on the other hand, information has until recently been scarce concerning how experimental manipulation of prevailing arterial pressure influence the in vivo pressure-distension relationships in precapillary vessels. To investigate whether arterial and/or arteriolar distension in healthy humans will decrease in response to iterative increments in local intravascular pressure, 11 individuals were subjected to a 5-week vascular pressure-habituation regimen (Eiken and Kölegård 2011). The pressure in the blood vessels of one arm was elevated during 40-min “habituation sessions”, executed 3 times/week. During the first week, the pressure elevation was 65 mmHg. It was then incremented in steps of 10 mmHg every week to 105 mmHg during the last week. The habituation regimen reduced pressure distension and pressure-induced flow in the brachial artery by 46 and 49 %, respectively (Fig. 4), effects that were completely reversed 4 weeks after the subjects finished the regimen and reassumed their normal daily routines. These findings demonstrate that intermittent, moderate intravascular pressure elevations over a few weeks are sufficient to markedly increase arterial and arteriolar stiffness in healthy humans. That the cumulated time of elevated pressure comprised merely 1 % of the 5-week habituation period, suggests that wall stiffness is determined by brief high-pressure episodes rather than the average pressure in the arteries/arterioles, and that the mechanisms regulating wall stiffness are remarkably sensitive to such episodic pressure elevations.

The mechanisms underlying pressure-induced increments in the wall stiffness of precapillary vessels are as yet not fully understood. It is clear that humoral mechanisms cannot account for the habituation effects since pressure distension decreases in the vasculature of the pressure-habituated arm but not in that of the unhabituated control arm (Eiken and Kölegård 1999, 2011). A principal question is whether iterative pressure loading increases the stiffness of local precapillary vessels by (1) increased smooth muscle tone resulting from increased myogenic responsiveness or increased local release of vasoactive substances, or by (2) increased passive elastic recoil resulting from altered structure of the vessel walls.

This was explored using the analysis technique described previously (Fig. 3) to determine whether the reduced pressure distension of arteries and arterioles, induced by the pressure-habituation regimen (Eiken and Kölegård 2011), consisted in elevation of the pressure threshold at which distension commenced or in attenuation of the gain for pressure distension. Although there were upward shifts of the thresholds, the reduced distensions at high pressures were predominantly attributable to blunted pressure-distension gains (Table 2). This might suggest that the 5-week habituation not only increased myogenic tone in arteries and arterioles but also induced structural changes in the walls of these vessels. Based on the time course for pressure-induced structural changes in the resistance vessels of rodents, it has been estimated that in humans, significant changes in such vessels would be apparent after several weeks and fully developed after a few months of pressure loading (Folkow 2010). That conventional ultrasound images did not detect any media hypertrophy or increased wall thickness-to-lumen ratio of the pressure-habituated brachial artery (Kölegård 2010), does not exclude that structural changes contributed to the increased arterial/arteriolar stiffness. Such imaging techniques might not detect minor changes in the intima-media thickness (cf. Schmidt and Wendelhag 1999); thus, in the aforementioned experiments, changes less than about 0.1 mm, corresponding to 20 % of the arterial wall thickness, might have passed undetected (Kölegård 2010). In addition, arterial ultrasound images are not capable of indicating if, or to what extent, repeated pressure exposures affect arteriolar wall thickness. Because flow is proportional to the fourth power of the lumen radius, an average concentric increase in arteriolar wall thickness of merely 12–13 % would suffice to explain the flow reduction induced by the pressure-habituation regimen. Finally, increased elastic recoil of a vascular wall may not be solely due to hypertrophy. Resistance vessels of hypertensive individuals commonly exhibit signs of eutrophic remodelling, characterized by increased wall thickness-to-lumen ratio with unaffected wall thickness and reduced lumen area (Folkow 1982; Mulvany 2002). Increased elastic recoil may also be a consequence of rearrangement of extracellular protein structures in the wall, including increased collagen-to-elastin ratio (Intengan et al. 1999a), and altered attachment of fibrillar components to the smooth muscle cells (Intengan and Schiffrin 2001). In vitro experiments and in vivo experiments in animals suggest that the pressure-induced changes in arterial/arteriolar wall structure develop in a sequential order. Depending on the magnitude and duration of the pressure loading, eutrophic remodelling may be followed by changes in fibrillar protein content/arrangement and by hypertrophy (Intengan et al. 1999b; Fridez et al. 2003). The pressure stimulus needed to induce such changes in human vessels is as yet not known.

**Table 2:** Pressure-diameter and pressure-flow relationships in the brachial and posterior tibial arteries in terms of pressure thresholds for diameter and flow increments, assumed to represent arterial and arteriolar distension thresholds, as well as the gains for the pressure-induced increments in these variables, as affected by 5 weeks of pressure habituation (PH) and bedrest (BR), respectively

	Brachial artery before 5 weeks PH	Brachial artery after 5 weeks PH	Tibial artery before 5 weeks BR	Tibial artery after 5 weeks BR
Diameter				
Threshold (mmHg)	190 ± 26	199 ± 23	250 ± 31	220 ± 37*
Gain (∆ %/mmHg)	0.31 ± 0.17	0.15 ± 0.10*	0.21 ± 0.16	0.33 ± 0.18*
Flow				
Threshold (mmHg)	185 ± 20	202 ± 41*	234 ± 16	230 ± 21
Gain (ml/mmHg)	6.3 ± 3.6	2.8 ± 2.6*	0.43 ± 0.35	0.83 ± 0.56*

* Denotes difference (*p* < 0.05) between before and after pressure habituation and bedrest, respectively. Threshold and gain values are based on results obtained from Eiken et al. (2008) and in Eiken and Kölegård (2011). Values are mean ± SD

Several substances that may be released locally in peripheral vascular beds in response to pressure-induced increase in shear stress or tension of the vessel walls (cf. Osol et al. 2002; Dekker et al. 2005) are capable both of acutely increasing the myogenic tone and of inducing long-term structural changes. Thus, elevation of pressure in the blood vessels of an arm by 90–150 mmHg induces local intravascular release of Angiotensin II (Ang-II) and endothelin-1 (ET-1) (Gustafsson et al. 2013), both being potent acute vasoconstrictors (Miyauchi and Masaki 1999; Mehta and Griendling 2007) as well as capable of stimulating smooth muscle growth and increase the media thickness-to-lumen ratio (Boonen et al. 1993; Moreau et al. 1997; Miyauchi and Masaki 1999; Dao et al. 2001; Mahmud and Freely 2002). The fact that typically only fractions of locally produced Ang-II and ET-1 spill over into the blood stream (Campbell 1987; Yoshimoto et al. 1991; Hilgers et al. 1998) suggests that the observed pressure-induced local intravascular release (Gustafsson et al. 2013) indeed reflected biologically relevant production of these substances in the pressurized vasculature. It appears that in humans, transient intravascular pressure increments may induce long-lasting local elevations of Ang-II and ET-1, and it can hence be assumed that repetitive pressure elevations may have cumulative effects on the local induction and release of Ang-II and ET-1 (Gustafsson et al. 2013).

Presumably, other local mechanisms are also involved in the adaptation of vascular wall stiffness to sustained, or iterative, changes in the intravascular pressure. Gravity-dependent changes in vascular responsiveness in long-term tail-suspended rats appear to be associated with altered availability of nitric oxide (NO) (Jasperse et al. 1999; Vaziri et al. 2000). Likewise, it has been shown that local NO-mediated dilatation of precapillary vessels is reduced in hypertensive patients (Muiesan et al. 2009). By contrast, in the study by Eiken and Kölegård (2011) flow-mediated vasodilatation was unaffected by the pressure habituation. This does, however, not exclude the possibility that the habituation regimen affected NO function, since the technique employed (Coretti et al. 2002) might not be sufficiently sensitive to detect minor changes in NO-dependent dilatory capacity (Sorensen et al. 1995). Notably, in the study by Gustafsson et al. (2013) the local vascular pressure provocation also led to a delayed plasma reduction of vascular endothelial growth factor A (VEGF-A), a potent vasodilator that is linked to the endothelial NO system; NO constitutes a downstream factor in the VEGF-A pathway as well as an upstream activator of VEGF-A expression (Tsurumi et al. 1997; Ziche et al. 1997). Considering that increased blood flow, resulting in increased vascular shear stress, is known to stimulate local release of both NO and VEGF-A (Baum et al. 2004), it may seem contradictory that marked regional elevations of vascular pressure and flow decreased plasma VEGF-A (Gustafsson et al. 2013). It is known, however, that the bioavailability of NO is reduced by Ang-II (Mehta and Griendling 2007). Consequently, it might be speculated that, under conditions of markedly increased vascular wall tension, any shear stress-induced stimulation of VEGF-A expression and NO release are counteracted by local production of Ang-II. The interplay between local vasodilatory and vasoconstrictor mechanisms is, however, largely unknown.

## Arterial pressure-distension and pressure-flow relationships as affected by long-term sustained reductions of transmural pressures

**Fig. 5:**
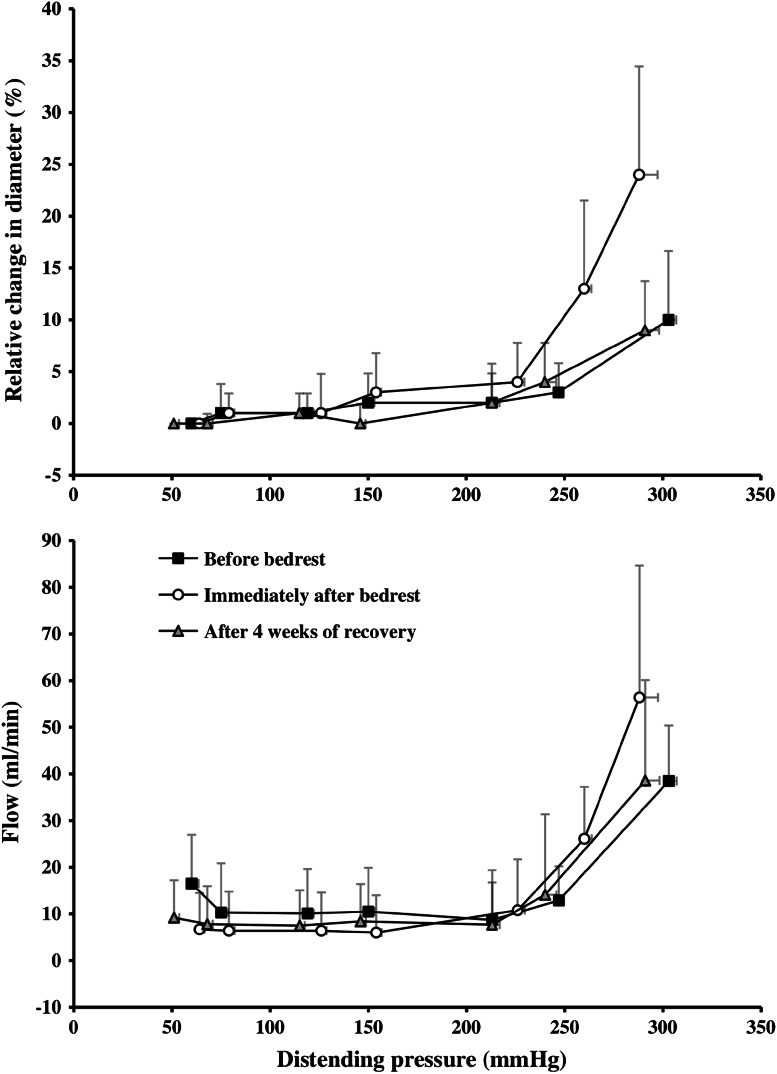
Relative change in the lumen diameter (*upper panel*) and flow (*lower panel*) in the posterior tibial artery as functions of distending pressure, before and immediately after a 5-week period of sustained horizontal bedrest and after 4 weeks of ambulatory recovery. Values are means (SD). *n* = 10. Adapted from Eiken et al. (2008)

The observation that the stiffness in arteries and arterioles is higher in the legs than in the arms in healthy individuals (Eiken and Kölegård 2004), does, as mentioned, not indicate if these differences are permanent or whether regular local pressure elevations, induced by assuming erect posture, or otherwise, are required to preserve the high stiffness in dependent vessels. To challenge the hypotheses that arterial and arteriolar stiffness will decrease in response to prolonged recumbency, and that such changes will be especially pronounced in the lower legs, 10 healthy subjects were exposed to 5 weeks of sustained bedrest in the horizontal position (Eiken et al. 2008). Bedrest increased pressure distension threefold in the tibial artery (Fig. 5) and merely by about a third in the brachial artery (Eiken et al. 2008). Also the pressure-induced increase in tibial artery flow was 2.8 times greater after bedrest, whereas the brachial artery flow response was unaffected by bedrest. The results indicate that elimination of the hydrostatic pressure gradients that act along the blood vessels in erect posture markedly increases pressure distension in arteries and arterioles of the legs, and that the higher wall stiffness observed in dependent arteries is not inherent but an acquired feature that needs to be maintained by regular exposures to gravity-induced pressure loading. Presumably, such reduction of the stiffness in precapillary vessels of the lower body contributes to the orthostatic intolerance occurring after a prolonged period in the recumbent position (Fortney et al. 1996). It should be noted that prolonged bedrest reduces the wall stiffness also in leg veins (Kölegård et al. 2009). Reduced stiffness in dependent veins may lead to excess pooling of blood in the lower body and hence to curtailed cardiac stroke volume when assuming erect posture. Thus, venous deconditioning may also contribute to the orthostatic intolerance following prolonged bedrest (Kölegård et al. 2009).

That the bedrest-induced decrements in lower-leg arterial/arteriolar stiffness was predominantly due to increased gain of the pressure-distension relationship, but only marginally attributable to a reduced threshold for pressure distension (Table 2), may suggest that the bedrest regimen led to structural changes in the walls of dependent arteries and arterioles (cf. Folkow and Sivertsson 1968; Folkow et al. 1970a, b; Folkow 1982). In a subsequent 5-week bedrest study, the intravascular pressure perturbations of daily life in an ambulatory upright posture were rather crudely mimicked in one lower leg by intermittently, throughout the bedrest, exposing the leg to a subatmospheric pressure of 90 mmHg (Eiken et al. 2011). It was found that local release of ET-1, induced by marked elevations of the transmural pressure, was less in the vasculature of the lower leg that had remained recumbent and inactive during the bedrest than in the pressure-conditioned vasculature of the contralateral leg. These results suggest that local down-regulations of the synthesis, and/or impaired release, of vasoactive substances take part in reducing wall stiffness of arteries and arterioles in response to reduced pressure loading. That fifteen 40-min exposures, during the 5-week bedrest, to a transmural pressure similar to that encountered in erect posture, constitutes a sufficient stimulus to prevent drops in arterial and arteriolar stiffness, complements the results from the pressure-habituation study (Eiken and Kölegård 2011), and underlines that the mechanisms regulating wall stiffness are highly sensitive to intermittent pressure elevations.

That bedrest reduced the pressure resistance of dependent arteries and arterioles (Eiken et al. 2008) is in good agreement with several experimental studies on rodents, in which the effect of local intravascular pressure unloading on the mechanical properties of precapillary vessels has been studied by surgically ligating selected arteries (Folkow et al. 1971, for review see Folkow 1982) or by suspending the animals with their back body and hindlimbs elevated (for review see Zhang 2001, 2013). Presumably, abolishing gravity-dependent hydrostatic pressure gradients in the circulatory system by exposure to microgravity will have similar effects on dependent arteries/arterioles. It can also be assumed that reduced stiffness in dependent resistance vessels contributes to the orthostatic intolerance experienced by astronauts after long-duration space missions (cf. Buckey et al. 1996). It is, therefore, somewhat surprising that Tuday et al. (2007) concluded that microgravity exposure leads to an overall increase in arterial stiffness that may contribute to the microgravity-induced orthostatic intolerance. Overall arterial stiffness was approximated by tracking dynamic changes in the diameter of the proximal aorta during the course of a pulse-pressure wave, while the subject was positioned supine (Tuday et al. 2007). It remains to be investigated how prolonged exposure to microgravity affects regional differences in the pressure-distension relationships of precapillary vessels.

## Plasticity of precapillary vessel stiffness; implications for circulatory control in healthy individuals and for the pathogenesis of primary hypertension

Evidence reviewed in the previous sections of this article, suggests that in healthy humans, the in vivo stiffness of peripheral arteries and arterioles constitutes highly plastic properties that adapt to cope with demands imposed by changes in the prevailing pressure, and that such adaptations are regulated by local mechanisms. The readiness by which the pressure-distension relationships of arteries and arterioles adapt to changes in the prevailing pressure, does per se suggest that precapillary wall stiffness constitutes a salient component of arterial pressure regulation, a notion that is corroborated by specific evidence. Thus, the observations that precapillary stiffness is higher in the lower leg than in the arm, and that the stiffness of the arm vessels does not suffice to preserve lumen diameters upon exposure to static distending pressures of the magnitude encountered by lower-leg vessels in erect posture (Eiken and Kölegård 2004), suggest that the elevated stiffness in leg vessels acts to regulate total blood-flow resistance and, therefore, to preserve pressure in central arteries in upright body positions. That a period of sustained recumbency reduces stiffness in dependent arteries and arterioles (Eiken et al. 2008) suggests that, even after life-long adaptation to intravascular pressure gradients associated with erect posture, precapillary vessels need regular exposure to such gradients to maintain their pressure resistance characteristics. Hence, decreased stiffness in dependent arteries/arterioles may contribute to the orthostatic intolerance observed in patients who have been bedridden for extended time periods (Lathers and Charles 1994; Fortney et al. 1996; Feldstein and Weder 2012), as well as in astronauts/cosmonauts returning to Earth’s gravity force field after prolonged spaceflights (Lathers and Charles 1994; Buckey et al. 1996).

That the adaptability of arterial/arteriolar stiffness constitutes an important factor in arterial pressure regulation is further suggested by the observation that the pressure resistance in precapillary leg vessels appears to markedly influence an individual’s capacity to maintain adequate arterial pressure at the head level, when dependent arteries/arterioles are exposed to critical distending pressures (i.e. >250 mmHg) during gradually increasing gravitoinertial load in the head-to-foot direction (Eiken et al. 2012). However, it remains to be established whether the relationship between the tolerance to high-G loads and the stiffness in dependent arteries/arterioles is causal; it is tempting to speculate that local arterial and arteriolar adaptation to the prevailing pressure load, contributes to the well recognized increase in G tolerance following repeated high-G exposures, i.e. G training (for review see Burton and Smith 1996).

That intermittent, moderate pressure elevations in the arm vessels, cumulatively amounting to a mere fraction of the total time period during which such pressure elevations are executed, induce marked stiffness increments in local precapillary vessels (Eiken and Kölegård 2011), suggests that arterial/arteriolar stiffness is determined by the brief episodes of increased pressure loading rather than by the average local intravascular pressure. The finding that increased intraarteriolar pressure leads to increased flow resistance reveals a positive feedback loop that, under conditions during which the pressure elevations are systemic rather than local, may constitute a prominent feature in the pathogenesis of primary hypertension, and is consistent with the notion that local pressure load acts as a prime mover in the development of vascular changes in hypertension (cf Folkow 1982, 1990, 2004; Dobrin 1983; Intengan and Schiffrin 2000). Nevertheless, whichever the mechanisms are that govern pressure-induced long-term increments in arterial and arteriolar stiffness, it appears that they are efficiently counteracted during physical exercise. Even though both dynamic and static muscular exercise induce marked, sustained arterial pressure elevations, chronic exercise does not induce hypertension. By contrast, regular aerobic training clearly reduces the risk of developing primary hypertension (for review see Hamer 2006). It remains to be elucidated if, and in what manner, aerobic exercise differs from other conditions associated with temporary elevations of arterial pressure, as regards how pressure-induced local vascular production of vasoconstrictors capable of stimulating smooth muscle growth, interact with local and systemic long-term vasodilatory mechanisms.
